# BENEFITS AND BARRIERS OF COMMUNITY-ENGAGED RESEARCH AND STRATEGIES FOR STRENGTHENING PARTNERSHIP PRACTICE

**DOI:** 10.1093/geroni/igad104.0275

**Published:** 2023-12-21

**Authors:** Carrie Leach, Rodlescia Sneed, Tam Perry

## Abstract

The meaningful engagement of older people in research may help to address their lag behind other groups in health outcomes and low participation in studies. This symposium addresses the critical gap in the aging literature and advances knowledge about community engagement by offering insight from experts on the complexities of partnership and engagement processes that vary by context as well as tools and tactics for overcoming obstacles. The first speaker will present findings and lessons gained from evaluating the needs of a self-organizing community advisory board of Black older adults to increase enrollment in a participant research pool. The second will focus on the challenges of centering a diverse range of partners in the design of a health and wellness program tailored to the needs of formerly incarcerated mid and late-life adults. The third speaker will provide recommendations for researchers interested in working with Indigenous communities and strategies for working collaboratively, and how to overcome relational challenges. The fourth speaker will present findings from a reflective evaluation process of a community-based participatory research partnership that evolved over seven years and discuss how the partnership evaluation tool can enhance partner practice. This symposium concludes with reflections about meaningful and authentic academic-community partnerships in line with supporting and building bridges between aspiring and existing community engaged scholars and older people who are experts on aging.

